# Cross-Linked Supramolecular Polyurea Elastomers with Mechanical Robustness and Recyclability

**DOI:** 10.3390/molecules30204061

**Published:** 2025-10-12

**Authors:** Yanping Li, Chong Wang, Bo Qin

**Affiliations:** 1 College of Information Science and Engineering, Changsha Normal University, Changsha 410100, China; 11526023@zju.edu.cn; 2 State Key Laboratory of Chemo and Biosensing, College of Chemistry and Chemical Engineering, Hunan University, Changsha 410082, China; wangchong6079@163.com

**Keywords:** cross-linked polymers, recyclable materials, hydrogen bonds, polyureas, supramolecular chemistry

## Abstract

Cross-linked polymers are indispensable in advanced applications, but suffer from poor recyclability due to permanent covalent networks. Herein, we report recyclable supramolecular polyurea elastomers that integrate ureidopyrimidinone-based quadruple hydrogen-bonding motifs directly into the polymer backbone. The dynamic and reversible nature of these motifs imparts the SPUEs with remarkable malleability and reprocessability while preserving the robustness of conventional polyureas. The SPUEs display remarkable mechanical robustness, solvent resistance, and facile reprocessability through hot-pressing, producing homogeneous films with minimal performance loss. Impressively, tensile strength, elongation at break, and toughness retained high recovery after reprocessing, demonstrating excellent closed-loop mechanical recyclability. This work showcases supramolecular engineering as a powerful strategy to reconcile mechanical robustness with recyclability in cross-linked polymers, offering new opportunities for sustainable thermosets and elastomers in circular materials design.

## 1. Introduction

Cross-linked polymers, including thermosets and elastomers, are indispensable in modern materials science, owing to their outstanding mechanical strength, chemical stability, and dimensional robustness [1]. Their permanent network structures confer high resistance to deformation and enable applications in coatings, adhesives, aerospace components, and structural composites. In recent years, the growing demand for materials that maintain high performance under extreme thermal, mechanical, or chemical conditions has further expanded the use of such systems in sectors such as flexible electronics, transportation infrastructure, and biomedical devices. However, the very feature that endows these materials with exceptional stability—their irreversible covalent cross-links—also presents a major drawback [2]. Once cured, covalently cross-linked networks cannot be readily reprocessed or reshaped, as the covalent bonds cannot be dissociated or reformed under conventional thermal or mechanical conditions [3,4,5]. Consequently, at the end of their service life, most cross-linked polymers are destined for disposal through landfilling or incineration, which not only squanders valuable resources, but also aggravates environmental pollution [6,7,8,9,10]. Developing recyclable cross-linked polymers that maintain the high performance of conventional systems thus remains an urgent challenge in the pursuit of sustainable materials solutions [11,12,13,14]. Previous advances in vitrimers, associative exchange networks, and dynamic covalent adaptable systems have provided important conceptual progress, but many of these approaches still suffer from limitations such as high activation temperatures, slow network rearrangement rates, or compromised mechanical integrity during recycling. These challenges underscore the necessity of integrating reversibility without diminishing robustness, particularly for applications where repeated processing or reshaping is desired.

Among the various high-performance cross-linked systems, polyureas occupy a unique position [15]. Polyurea elastomers are widely used in protective coatings, elastomers, adhesives, and sealants due to their remarkable mechanical robustness, thermal stability, and resistance to chemicals and solvents [8,16,17,18]. Commercial formulations are typically produced by the rapid reaction of isocyanates with long-chain polyether amines and small-molecule chain extenders and cross-linkers. The resulting polyurea networks are heavily reinforced by dense arrays of stable and robust urea linkages and extensive intermolecular hydrogen bonding [19,20]. These strong cohesive interactions are crucial for sustaining load-bearing capacity and enhancing durability under cyclic deformation, which explains their prominence in industrial-scale applications. While these interactions contribute to their outstanding durability, they also impart stable characteristics of cross-linked polymers that preclude melt processing or recycling. Overcoming this intrinsic limitation without sacrificing mechanical performance has therefore emerged as a pressing research frontier [21,22,23,24].

One promising approach is the incorporation of dynamic, reversible supramolecular motifs into polyurea backbones [21,23]. Supramolecular chemistry has offered powerful strategies to endow otherwise rigid polymers with adaptive properties, where noncovalent interactions such as hydrogen bonding, π-π stacking, metal–ligand coordination, or host–guest recognition can act as dynamic chain-extending or cross-linking points [25,26,27,28,29,30,31,32,33,34,35,36]. Notably, several pioneering reports have highlighted how multiple hydrogen-bonding motifs can endow polyurea networks with enhanced stress relaxation, shape-memory behavior, and thermal reprocessability while retaining structural integrity. These systems often exhibit impressive recyclability under hot-pressing or solvent-assisted conditions and maintain performance across multiple reprocessing cycles, indicating the feasibility of supramolecular design in practical settings. In particular, multiple hydrogen-bonding units can introduce reversible, yet strong, interactions that mimic the role of permanent covalent bonds while retaining the ability to undergo bond exchange. By carefully balancing the reversibility and stability of these motifs, it is possible to construct cross-linked networks that combine high mechanical performance with recyclability [20,37,38,39,40,41,42,43,44,45,46]. Quadruple hydrogen-bonding motifs represent one of the most effective supramolecular interactions for this purpose. Ureidopyrimidinone (UPy) dimers, for example, are well known for their high binding affinity (association constants up to 10^7^ M^−1^), directional specificity, and excellent self-complementarity [47,48,49,50]. Moreover, the modular nature of UPy units enables their integration into both hard and soft segments of polymer backbones, providing tunable network densities and adjustable relaxation times. This versatility facilitates the design of materials that can respond to moderate heating, pressure, or solvent exposure, thereby offering routes to closed-loop recycling and reshaping without extensive chemical degradation. These features render UPy units ideal candidates as reversible cross-linkers in polymer networks. When incorporated into polyurea chains, UPy groups can form dynamic supramolecular arrays that temporarily dissociate and reassemble under thermal or mechanical stimuli. Such dynamic behavior not only enables stress relaxation and topological rearrangement, but also facilitates reprocessing through hot-pressing or reshaping, while cooperative interactions with urea linkages preserve the material’s mechanical integrity [51,52].

**Figure 1:**
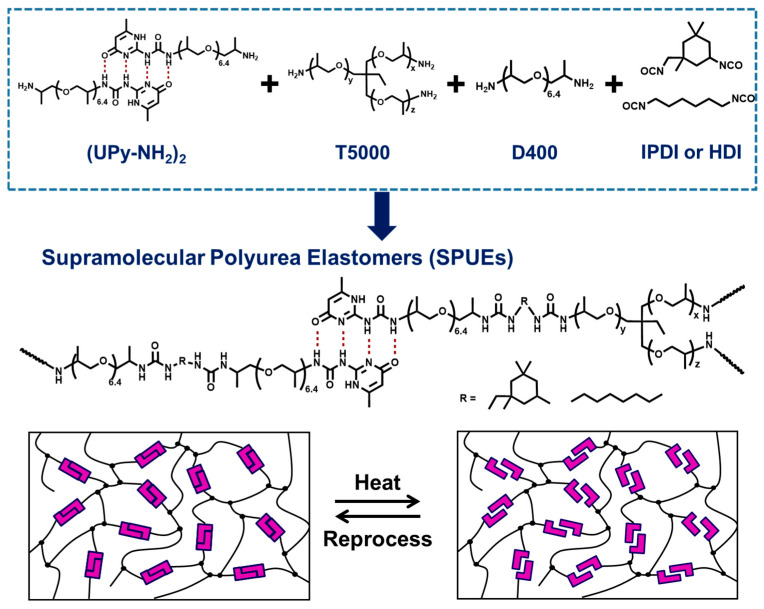
The design and synthesis of cross-linked supramolecular polyurea elastomers (SPUEs) and a schematic diagram of the reprocessing process of SPUEs based on the strategy of incorporating noncovalent bonds.

Herein, we report the design and synthesis of recyclable cross-linked supramolecular polyurea elastomers (SPUEs) that integrate UPy-based quadruple hydrogen-bonding motifs directly into the polymer backbone (Figure 1). This molecular engineering strategy yields elastomers that combine the robustness of conventional polyureas with unprecedented recyclability. The SPUEs exhibit remarkable mechanical strength and solvent resistance, while also demonstrating stress relaxation and thermal reprocessability under mild mechanical recycling conditions. Notably, the materials maintain dimensional stability and show minimal degradation in tensile properties even after several processing cycles, reflecting the synergistic interplay between supramolecular cross-links and intrinsic urea domains. Importantly, the dynamic exchange of UPy hydrogen bond arrays provides a fine balance between structural adaptability and network stability, enabling the preservation of mechanical performance over multiple reprocessing cycles. In contrast to traditional thermosetting systems, the present design minimizes waste generation and allows for reshaping into new configurations with negligible loss of function. Such characteristics offer a relevant platform for future integration into scalable manufacturing and circular polymer value chains. This work establishes a new design paradigm for sustainable cross-linked polymers by showing that supramolecular engineering can reconcile mechanical robustness with recyclability—two features traditionally viewed as mutually exclusive. Beyond polyurea elastomers, the present strategy highlights a general approach for the development of next-generation recyclable thermosets and elastomers, offering promising opportunities for advancing circular polymer economies and addressing the growing challenges of polymer waste management. Looking forward, the principles demonstrated here may be extended to other classes of covalent networks, such as polyurethanes, epoxies, or polyimides, where the integration of dynamic supramolecular bonds could enable high-value reuse and upcycling without undermining structural performance.

## 2. Results and Discussion

### 2.1. Preparation and Characterization of SPUEs

**Figure 2:**
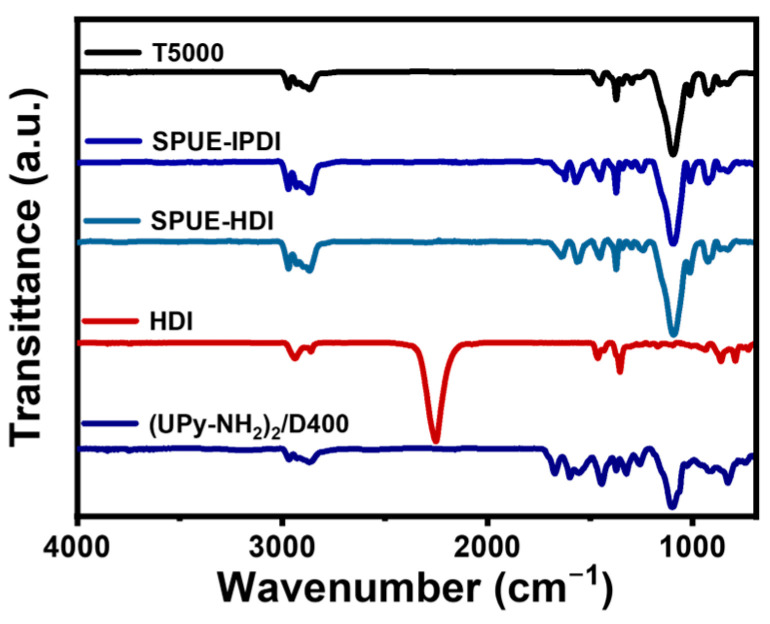
FT-IR spectra of SPUE-IPDI and SPUE-HDI, diamine monomers including (UPy-NH_2_)_2_/D400, triamine monomer T5000, and diisocyanate monomer.

The successful synthesis of SPUEs was verified by FT-IR spectroscopy. As shown in Figure 2, the characteristic absorption of the isocyanate groups (-NCO, 2260 cm^−1^) completely disappeared compared to the diisocyanate monomer, confirming nearly quantitative conversion of the isocyanate-amine reaction. Concurrently, the emergence of new bands at 3330 and 1640 cm^−1^, corresponding to -NH- and C=O stretching vibrations of urea linkages, further substantiated polyurea formation. Similar spectral features were observed for SPUE-HDI samples with different isocyanate monomers. Therefore, these results provide evidence that SPUEs bearing quadruple hydrogen bonds were successfully obtained via a highly efficient isocyanate-amine coupling strategy.

### 2.2. Mechanical and Thermal Performance of SPUEs

**Figure 3:**
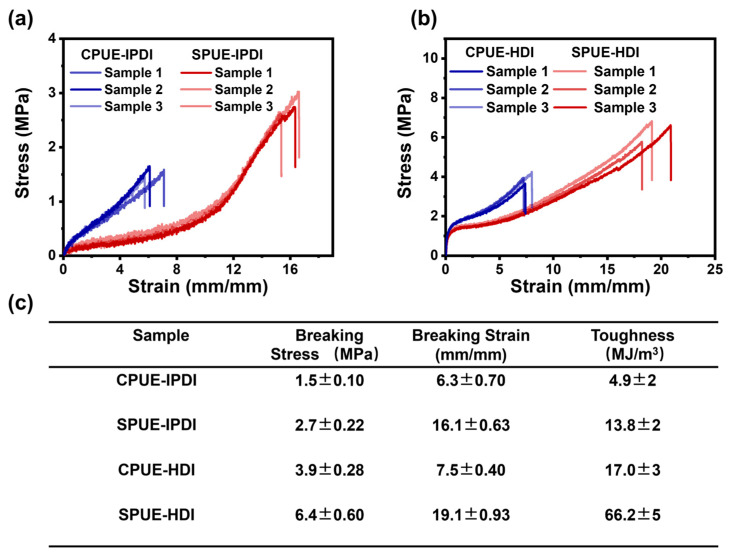
(**a**) Stress–strain curves of CPUE-IPDI and SPUE-IPDI. (**b**) Stress–strain curves of CPUE-HDI and SPUE-HDI. (**c**) The mechanical properties, including the breaking strength, breaking elongation, and the toughness of the above polyurea elastomers.

To elucidate the effect of noncovalent interactions and hard-segment chemistry on mechanical performance, the specimens of SPUEs were fabricated by hot-pressing and were subsequently evaluated by uniaxial tensile testing at a strain rate of 10 mm·min^−1^ under ambient conditions. The representative stress–strain curves clearly demonstrate that the mechanical properties of polyurea elastomers can be effectively tuned by varying both the content of UPy-based supramolecular motifs and the type of diisocyanates employed (Figure 3a,b). Specifically, incorporation of the noncovalently bonded UPy units markedly enhanced ductility: the breaking elongations of SPUE-IPDI and CPUE-IPDI reached 630% and 1610%, respectively, whereas SPUE-HDI and CPUE-HDI exhibited even higher elongations of 750% and 1910%. Concomitantly, the tensile strength was significantly reinforced, with CPUE-IPDI, SPUE-IPDI, CPUE-HDI, and SPUE-HDI achieving values of 1.5, 2.7, 3.9, and 6.4 MPa, respectively. Although the Young’s modulus of SPUEs decreased slightly upon incorporation of supramolecular units, the toughness of SPUE-IPDI and SPUE-HDI was 13.8 and 66.2 MJ/m^3^ (Figure 3c), respectively, which is substantially improved compared with the covalent polyurea elastomers. These enhancements are probably attributed to the synergistic contribution of dynamic UPy motifs, which effectively dissipate stress through reversible bond exchange, and rigid hard segments derived from different diisocyanates. Collectively, these results unambiguously demonstrate that introducing noncovalent crosslinks in combination with tailored hard-segment chemistry significantly elevates the mechanical robustness of cross-linked polyurea elastomers.

**Figure 4:**
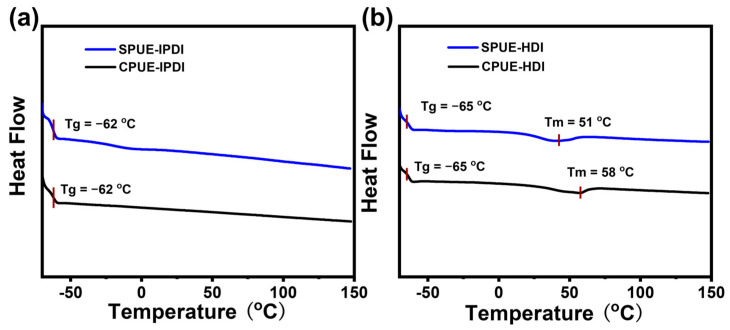
(**a**) DSC curves of CPUE-IPDI and SPUE-IPDI. (**b**) DSC curves of CPUE-HDI and SPUE-HDI.

**Figure 5:**
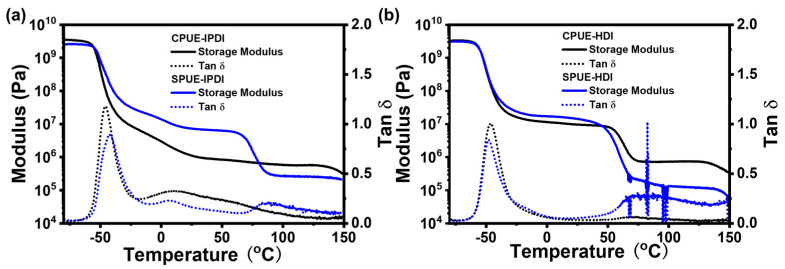
(**a**) DMA curves of CPUE-IPDI and SPUE-IPDI. (**b**) DMA curves of CPUE-HDI and SPUE-HDI.

To investigate the thermal characteristics of SPUEs, dynamic mechanical analysis (DMA) and differential scanning calorimetry (DSC) were conducted. As shown in Figure 4, the glass transition temperatures (Tg) obtained from DSC for IPDI-based and HDI-based polyurea elastomers were −62, and −65 °C, respectively, suggesting that incorporation of supramolecular motifs does not significantly alter the intrinsic rigidity of the soft segments of polymer networks. Notably, while IPDI-based polyurea elastomers (CPUE-IPDI and SPUE-IPDI) exhibited no discernible melting transitions, SPUE-HDI and CPUE-HDI displayed well-defined melting points at 51 and 58 °C, respectively, which can be attributed to the more regular packing of HDI-derived rigid segments. The results agree well with the higher Young’s modulus of HDI-based elastomers than DI-based elastomers. The consistently low Tg values (<−60 °C) indicate that SPUEs maintain typical elastomeric behavior at ambient temperature, owing to the flexibility of their soft-chain domains. In addition, DMA results also show clear glass transitions in low-temperature regions (~−50 °C) and exhibited typical plateaus in the storage moduli above the Tg indicated by DMA (Figure 5a,b), as expected for cross-linked polymeric networks. Therefore, SPUEs exhibit tunable thermal performance through introducing noncovalently bonded supramonomers and varying monomeric structures.

### 2.3. Solvent Resistance of SPUEs

**Figure 6:**
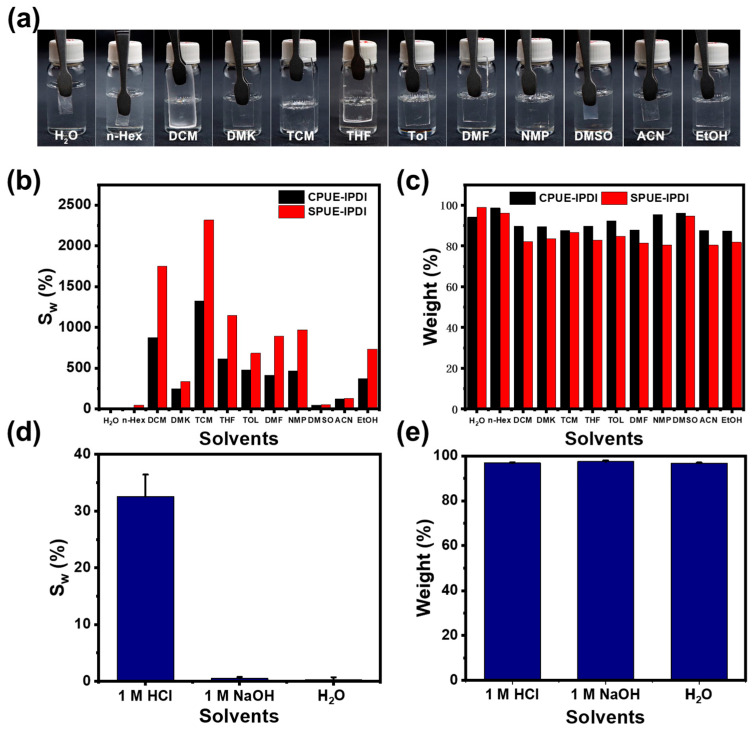
(**a**) Digital photos of SPUE-IPDI before and after soaking in different solvents for 24 h. (**b**) Mass swelling ratio of SPUE-IPDI in different solvents. (**c**) Gel fraction of SPUE-IPDI in different solvents. (**d**) Mass swelling ratio of SPUE-HDI in different aqueous environments. (**e**) Gel fraction of SPUE-HDI in different aqueous environments.

The solvent resistance of the supramolecular polyurea elastomers (SPUEs) was evaluated by immersing rectangular specimens for 24 h in a wide range of solvents, covering both polar and non-polar media. The solvents tested included water (H_2_O), ethanol (EtOH), acetonitrile (ACN), n-hexane (n-Hex), dimethyl sulfoxide (DMSO), dichloromethane (DCM), acetone (ACE), *N*,*N*-dimethylformamide (DMF), chloroform (TCM), toluene (TOL), and tetrahydrofuran (THF). Taking SPUE-IDPI as a representative example (Figure 6a,b), all samples retained their original macroscopic shape and exhibited only moderate swelling, with no significant signs of dissolution. This exceptional tolerance toward diverse solvents highlights the robustness of the cross-linked supramolecular networks and the structural stability endowed by the dynamic quadruple hydrogen-bonding motifs. Due to its relatively low cross-linking density compared with CPUE-IPDI, SPUE-IPDI displayed slight dissolution in a few solvents (Figure 6c). Importantly, SPUE-IPDI also showed excellent resistance to aqueous environments of varying pH, including acidic, alkaline, and neutral aqueous solutions (Figure 6d,e). After soaking in water for 24 h, the sample maintained similar tensile curves, with only minor reductions in tensile strength and elongation. Collectively, these results demonstrate that SPUEs incorporating quadruple hydrogen-bonding motifs possess remarkable network integrity and solvent resistance across a wide range of conditions, thereby underscoring their potential as durable and recyclable elastomeric materials.

### 2.4. Dynamic Properties and Recyclability of SPUEs

**Figure 7:**
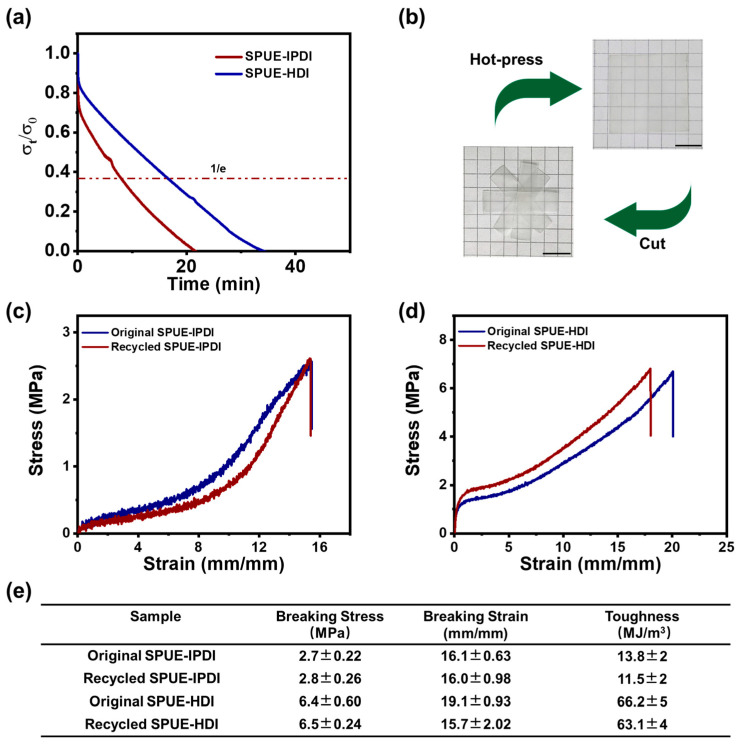
(**a**) Stress relaxation of SPUE-IPDI and SPUE-HDI at 120 °C using the DMA method. (**b**) Digital photos of SPUE-HDI before and after hot-pressing at 120 °C and 6 MPa for 10 min. (**c**) The stress–strain curves of SPUE-IPDI before and after mechanical recycling. (**d**) The stress–strain curves of SPUE-HDI before and after mechanical recycling. (**e**) Mechanical properties, including tensile strength, breaking elongation, and toughness of the original and recycled SPUEs.

The dynamic exchange of quadruple hydrogen bonds and urea linkages within the cross-linked polyurea networks endows the SPUE materials with notable malleability and reprocessability. To evaluate these dynamic characteristics, stress relaxation tests were performed on the SPUE networks using DMA. As shown in Figure 7a, the samples underwent rapid network reconfiguration and pronounced stress relaxation at elevated temperatures (e.g., 120 °C). The characteristic relaxation times of SPUE-IPDI and SPUE-HDI were determined to be 9.7 and 16.6 min, respectively, with the faster relaxation of SPUE-IPDI likely attributed to its amorphous microstructure. These results indicate that the stress relaxation behavior of the SPUEs originates from the thermally activated exchange of quadruple hydrogen bonds, thereby enabling efficient reprocessing and mechanical recycling.

Building on these insights, the reprocessability of SPUE was further evaluated. In a representative procedure, bulk SPUE-IPDI was cut into small fragments and hot-pressed at 120 °C under 6 MPa for 10 min. This simple operation produced a transparent, homogeneous film (Figure 7b), underscoring the excellent thermoplastic recyclability of the cross-linked networks. In contrast, after the recycling process, covalent cross-linked polyurea cannot be reprocessed to an integrate film due to the permanent cross-linked structures. To assess the structural integrity and performance retention of the reprocessed materials, tensile testing was conducted. Remarkably, the stress–strain profiles of the reprocessed SPUEs (SPUE-IPDI and SPUE-HDI) nearly overlapped with those of the pristine sample (Figure 7c,d), signifying minimal deterioration in mechanical performance, even after multiple recycling cycles. Quantitatively, the recovery of tensile strength, elongation at break, and toughness all exceeded 92% (Figure 7e), outperforming other reported dynamic recyclable elastomers [46]. These findings unambiguously demonstrate that the cooperative dynamics of quadruple hydrogen bonds preserve both network architecture and mechanical robustness, thereby enabling efficient closed-loop mechanical recycling.

## 3. Materials and Methods

### 3.1. Materials

2-Amino-4-hydroxy-6-methylpyrimidine, *N*,*N*’-carbonyldiimidazole (CDI), isophorone diisocyanate (IPDI), and hexamethylene diisocyanate (HDI) were purchased from Anhui Zesheng Technology Co., Ltd. (Hefei, China). Trimethylolpropane tri(polypropylene glycol) ether (amine terminated) (Polyetheramine T5000, M_n_ ≈ 5000 g/mol) was purchased from Shanghai Macklin Biochemical Technology Co., Ltd. (Shanghai, China). CDCl_3_ and DMSO-d_6_ were purchased from Shanxi Didu Medical Chemical Co., Ltd. (Xi’an, China). Polyetheramine D-400 (M_n_ ≈ 400 g/mol) was purchased from Zhangjiagang Top Chemical Co., Ltd. (Zhangjiagang, China). The organic solvents used for the solvent resistance test (analytical grade) were purchased from Guangdong Wanxin Petrochemical Technology Co., Ltd. (Zhongshan, China). All chemicals were used as received without further purification.

### 3.2. Synthesis

#### 3.2.1. Synthesis of the Supramolecular Diamine Monomer (UPy-NH_2_)_2_

2-Amino-4-hydroxy-6-methylpyrimidine (5.00 g, 0.040 mol) and CDI (9.00 g, 0.056 mol) were dissolved in 100 mL of DMSO in a 250 mL two-neck round-bottom flask. The mixture was stirred at 80 °C, during which the reactants gradually dissolved and a white precipitate formed. After 2 h, the reaction mixture was filtered, and the solid was washed thoroughly with acetone and dried for 3 h to afford Compound 1 as a white powder (5.46 g, yield ≈ 62%).

Next, compound 1 (5.00 g, 0.023 mol) and polyetheramine D-400 (30.8 g, 0.068 mol) were charged into a 250 mL two-neck flask under nitrogen and stirred at 40 °C for 15 h. The initially viscous suspension gradually became a homogeneous and transparent solution. The obtained product is a mixture of the supramolecular diamine monomer (UPy-NH_2_)_2_ and excess D-400, which was directly used for polymerization without further separation. The content of the supramolecular diamine monomer in the mixture was determined by ^1^H NMR spectroscopy. The integration of characteristic-peak methyl protons of the main chain and protons of the ureidopyrimidinone ring was used to calculate the relative molar fraction of (UPy-NH_2_)_2_. The supramolecular monomer content was estimated to be approximately 21%.

#### 3.2.2. Synthesis of Supramolecular Polyurea Elastomers (SPUEs)

The synthetic route is shown in Figure 1. A mixture containing supramolecular monomer/D-400 (3.0 g, 5 mmol) and covalent polyetheramine cross-linker T5000 (16.25 g, 3.25 mmol) was dissolved in toluene (5 mL of dichloromethane was added to facilitate dissolution). This solution was combined in a PTFE beaker which was purchased from Chongqing Xinwei’er Glass Co., Ltd. (Chongqing, China). with a toluene solution of HDI (1.66 g, 9.88 mmol) under vigorous stirring. A colorless and transparent gel formed within 10 min, and the reaction proceeded for another 50 min at room temperature. The product was filtered, washed three times with toluene, and dried at 60 °C under vacuum for 24 h to remove solvents and give SPUEs. A fully covalent polyurea reference was prepared analogously. SPUE granules were hot-pressed into films under the following conditions: pre-pressing for 1 min in a 45 × 45 × 0.4 mm mold at 150 °C and 5 MPa, followed by hot-pressing for 10 min in a 45 × 45 × 0.25 mm mold, and a second pressing for an additional 10 min. The resulting films were homogeneous and transparent.

### 3.3. Characterization

^1^H NMR spectra were recorded on a Bruker AV-III-400 spectrometer (Billerica, MA, USA, 400 MHz) using CDCl_3_ or DMSO-*d*_6_ as solvents. The stress–strain curves were measured by using a universal testing machine (SANS EUT6502 electric) supplied by Shenzhen Sansi Testing Technology Co., Ltd., Shenzhen, China at room temperature. For the tensile test, the polyurea samples were cut into rectangle shape (15 mm × 5 mm × 0.4 mm). The rate of extension was fixed at 5 mm min^−1^ for tensile tests. DSC measurements were performed under nitrogen atmosphere using a TA instrument Discovery 250 system (New Castle, DE, USA). All the samples (~5 mg) were heated from −70 to 150 °C with a rate of 10 °C/min. Data from the second heating cycle were used to determine the transition temperatures of materials. DMA of SPUEs was conducted on a TA instrument Discovery 850 system. The rectangular samples were heated from −80 to 150 °C with a heating rate of 3 °C/min and a constant frequency of 1 Hz. The preload force and amplitude were fixed at 0.01 N and 20 µm, respectively. To determine the solvent resistance of SPUEs, rectangular-film specimens were weighed and immersed in various organic solvents at room temperature for 24 h. After removal, excess solvent was blotted off and the specimens were reweighed. The samples were then dried for 24 h and weighed again. Mass swelling ratios and mass retention ratios were calculated to evaluate solvent resistance. Fragments of SPUE films (~0.6 g) were placed in a 45 × 45 × 0.25 mm mold and hot-pressed at 150 °C and 5 MPa for 10 min. After cooling and demolding, the films were re-pressed once more under identical conditions to improve uniformity. The reprocessed films were cut into rectangular strips for tensile testing, and their mechanical properties (tensile strength, elongation at break, and Young’s modulus) were compared with those of the original films. The Young’s modulus was calculated from the slope of the initial linear region (strain < 2%) of the stress–strain curve. The toughness was determined by integrating the area under the stress–strain curve.

## 4. Conclusions

In this work, we developed recyclable cross-linked supramolecular polyurea elastomers (SPUEs) by incorporating UPy-based quadruple hydrogen-bonding motifs into polyurea backbones. This molecular engineering strategy endows the SPUE networks with a unique combination of robustness and recyclability, reconciling two properties that were traditionally considered incompatible. The resulting SPUEs exhibit remarkable mechanical robustness, solvent resistance, and efficient reprocessability under mild hot-pressing conditions. Notably, the reprocessed materials retain high recovery of their tensile strength, elongation, and toughness, underscoring the structural integrity of the dynamic supramolecular networks. Given their excellent elasticity, toughness, and dynamic reversibility, these recyclable SPUEs also hold strong potential for use in flexible electronic devices, wearable sensors, and shock-absorbing or vibration-damping components, where material recovery and reconfigurability are highly desirable. The integration of covalent and noncovalent interactions not only enables the recyclability of supramolecular polyurea networks, but also offers a broadly applicable platform for extending this strategy to other high-performance cross-linked systems such as polyurethanes, epoxy resins, and rubbers. This approach provides a promising pathway for addressing the recycling challenges and environmental impacts associated with discarded thermoset polymers.

## Data Availability

All data are available in the manuscript.
